# Callous-unemotional traits, low cortisol reactivity and physical aggression in children: findings from the Wirral Child Health and Development Study

**DOI:** 10.1038/s41398-019-0406-9

**Published:** 2019-02-11

**Authors:** Nicola Wright, Jonathan Hill, Andrew Pickles, Helen Sharp

**Affiliations:** 1 Biostatistics Department at the Institute of Psychiatry, King’s College London, London, UK; 2 School of Psychology and Clinical Language Sciences, University of Reading, Reading, UK; 3 Department of Psychological Science, Institute of Psychology, Health and Society, University of Liverpool, Liverpool, UK

## Abstract

Callous-unemotional (CU) traits are thought to confer risk for aggression via reduced amygdala responsivity to distress cues in others. Low cortisol reactivity is thought to confer risk for aggression via reduced arousal and this effect may be confined to boys. We tested the hypothesis that the association between childhood CU traits and aggression would be greatest in the absence of the inhibitory effects of cortisol reactivity, and that this effect would be sex dependent. Participants were 283 members of a stratified subsample within an epidemiological longitudinal cohort (WCHADS). Cortisol reactivity to a social stressor was assessed at 5 years. CU traits were reported by mothers at 5 years, and physical aggression by mothers and teachers at age 7. Results showed that CU traits were associated with elevated aggression at 7 years controlling for earlier aggression. There was no main effect of cortisol reactivity on regression. The association between CU traits and aggression was moderated by cortisol reactivity (*p* = .011) with a strong association between CU traits and aggression in the presence of low reactivity, and a small and non-significant association in the presence of high reactivity. This association was further moderated by child sex (*p* = .041) with the joint effect of high CU traits and low cortisol reactivity seen only in boys (*p* = .016). We report first evidence that a combined deficit in inhibitory processes associated with CU traits and low cortisol reactivity increases risk for childhood aggression, in a sex-dependent manner.

## Introduction

Neurobiological models of persistent antisocial behaviours in children propose a prominent role for low physiological arousal leading to failures to inhibit aggressive behaviour, greater sensation seeking, and reduced effects of punishment^1,2^. Callous-unemotional (CU) traits are similarly thought to be associated with failures to inhibit aggression and reduced punishment learning, via mechanisms that implicate reduced amygdala activation to distress in others^3,4^. No previous study has examined the roles of both CU traits and reduced physiological arousal indexed by hypothalamic-pituitary-adrenal (HPA) axis activity in the generation of childhood aggression. Further, the effects may differ in males and females, with failures of inhibition contributing to aggression in boys, and heightened reactivity in girls^5–10^. Using cortisol reactivity to hearing a recorded argument between adults as the index of physiological arousal, we set out to test the hypothesis that the association between CU traits and aggression would be strengthened in the presence of low cortisol reactivity in boys but not girls^11,12^.

Several mechanisms have been proposed linking under-arousal to aggressive and antisocial behaviours notably via sensation-seeking and risk-taking behaviours^13^, and fearlessness with reduced inhibition of aggression^14,15^. These models would lead to the prediction that reduced HPA axis reactivity will be associated with more behaviour problems. Current evidence is however inconsistent perhaps explained in part by marked heterogeneity of child ages, study designs, methods for assessing cortisol, and symptom measurement^16^. The majority of studies of HPA axis functioning have examined contributions to broad externalising problems. However, biological mechanisms may vary within this broad phenotype. In recent years the construct of CU traits has proved robust and informative at identifying a subgroup of antisocial children who show more severe and persistent antisocial behaviour^17^. Thus there may be a distinct aetiology for antisocial behaviour, and in particular for physical aggression, associated with CU traits compared to antisocial behaviour without CU traits^18,19^. Deficits in recognition of fear and sadness, possibly related to lack of eye contact in social interactions, are thought to underpin indifference to others’ distress in CU traits, and hence to children’s failures to inhibit aggressive or cruel actions^3,20,21^.

Thus according to both the underarousal and CU traits hypotheses antisocial behaviours arise from failures in inhibitory mechanisms, and a lack of responsiveness to others’ behaviours, either punishment or to emotional distress. The greatest risk for antisocial behaviours, and in particular for physical aggression, may therefore arise from the combination of the two. Neurobiological models of CU traits implicate lack of responsiveness of the amygdala to emotional cues which would typically serve to inhibit antisocial behaviour^21,22^, with the possibility of reciprocal effects between HPA axis regulation and amygdala function^23,24^. These considerations led Hawes et al. to speculate that, “…high levels of CU traits and HPA-axis hypoactivity characterise a particularly severe subgroup.” Whether processes associated with CU traits and HPA axis hypo-activity are sufficiently distinct to make independent contributions to antisocial behaviours is not yet clear. Indeed it has been proposed that HPA axis hypoactivity contributes to CU traits, or that they are separate markers of the same underlying processes. However, studies examining whether CU traits are associated with HPA axis hyporeactivity have yielded inconsistent findings, and no studies have provided evidence to link reduced cortisol reactivity to CU traits outside of clinically referred samples^25^.

It has previously been proposed that the under-arousal pathway to antisocial behaviour problems may be characteristic of their development in boys but not in girls^12^. Increasingly there is evidence for sex differences in HPA axis mechanisms for antisocial behaviour in children, consistent with this hypothesis. This has been demonstrated in cross section and over time in a study 1768 children aged 10–12^5,7^, and in further cross-sectional studies of 245 adolescents^9^ and 501 adolescents^26^. In a longitudinal study of 283 children over the ages 6–9 more blunted cortisol rhythms at age 6 (less change across the day from morning to evening) predicted a greater increase in conduct problems and aggressive behaviour, more in boys than in girls^27^.

In summary, and with caveats arising from inconstancies in approaches and findings, available evidence is consistent with there being a pathway to aggression characterised by physiological underarousal and by lack of distress responsivity, which is more typical of boys than girls. In line with Hawes’ et al. speculation we predicted the association between CU traits and aggression will be greatest in the presence of low cortisol reactivity, and that that this would be seen in boys but not in girls. We tested these predictions prospectively in community-based sample of children, with cortisol reactivity and CU traits assessed at 5 years, and child aggression at age 7 years.

## Method

### Sample

Participants were members of the Wirral Child Health and Development Study (WCHADS), a prospective epidemiological study starting in pregnancy. Ethical approval was granted by the Cheshire North and West Research Ethics committee on the 27 June 2006 (pregnancy to age 1 waves), 7 June 2010 (age 2.5–5 year waves), and 22 December 2014 (age 7 and 9 year waves). All women gave written informed consent at the point of recruitment in the antenatal clinic. The study used a two stage stratified design in which a consecutive general population sample (the ‘extensive’ sample) is used to generate a smaller ‘intensive’ sample stratified by psychosocial risk with more detailed measurement over time and both are followed in tandem^28,29^. [Further information about the data and conditions for access are available at the University of Liverpool Research Data Catalogue: 10.17638/datacat.liverpool.ac.uk/564.]

The whole cohort (extensive sample) comprised 1233 women recruited in pregnancy with a live, singleton baby for long-term follow-up post-birth (see Sharp et al.^28^ for detailed account of sampling). The mean age at recruitment was 26.8 years (SD = 5.8, range 18–51), 41.8% of the sample were in the most deprived quintile of UK neighbourhoods^30^ and 96.1% were White British. There were 316 mothers recruited to the intensive sample at 32 weeks pregnancy and available at birth for longitudinal follow-up. At age 5 a further stratum was added to the intensive sample using established thresholds for emotional or behavioural symptoms in the Child Behaviour Checklist (CBCL^31^) and the Antisocial Process Screening Device (APSD^32,33^) used with the extensive sample at age 3.5 years. This identified 94 children of whom 75 (79.8%) agreed and completed the age 5 assessment. This process yielded an intensive sample of 330 at age 5, of whom 314 (90%) provided complete cortisol data. Eight cases were subsequently excluded; five had supra-physiological levels of cortisol and three had eaten or drunk prior to the final sample being taken.

In the analyses that follow, data from the larger extensive sample assessed at 7 (*n* = 778) were used to first estimate the aggression latent variable outcome variable from the mother (*n* = 769) and teacher reports (*n* = 725). The main analyses then used data from the intensive sample comprising 283 cases (of the 330 available from age 5) who provided data at both age 5 and 7 years. The mean age of this sample at the age 5 assessment was 57.59 months (SD = 2.44, range 54–69) and at the age 7 assessment was 88.19 months (SD = 3.75, range = 83–107) with slightly more boys (*n* = 145) than girls (*n* = 138). Of these 283, 15.9% (*n* = 45) of children (62% boys) showed clinically significantly externalising problems on the CBCL according to mother or teacher report.

### Code availability

Analysis code is available from the first author.

### Procedures and measures

#### Age 5 procedures and measures

At age 5 the families in the intensive sample completed a 2.5 h lab assessment within which the stress induction task was embedded. Because arrival at the lab may activate an inticipatory cortisol rise, Koss and Gunnar^34^ recommend a 30–40 min relaxation period prior to taking a baseline sample. In this study, it was not possible to ensure this period was relaxing because in order to complete all the assessments it was necessary to present a range of cognitive and emotion recognition tasks to the children during the first 40 min. Therefore, to provide a more robust baseline we took saliva samples at 20 and 40 min and used an average of the two. The child was exposed to the stress induction paradigm followed by an emotionally neutral cognitive task, with the post-stress cortisol sample taken 20 min after onset of the argument. Researchers’ ensured that the child had been awake and had not eaten or drank for the 30 min prior to the first sample. Mothers were briefed about the stress induction task in private and children were debriefed after the procedure.

#### Stress induction paradigm

The stress paradigm involved the child overhearing an audiotaped recording of an argument between two adults^35^. The task been used in previous studies of respiritory sinus arrhythmia^35–37^ and galvanic skin response^35,38^. Mothers were asked to wait behind a screen whilst the child remained in the lab with the researcher completing the Kiddie Connors Continuous Performance Task^39^. The recorded conversation started playing 15 s into the task, after a few seconds the researcher informed the child that the sound was people speaking in the next room, the researcher then sat away from the child and busied themselves with paperwork for the remainder of the recording. The 7 min recording comprised 2 min in which two work colleagues could be heard chatting about benign topics, 2 min intense argument, 2 min unresolved anger, and 1 min verbal resolution.

#### Salivary hormone assessment and enzyme immunoassay procedure

Salivary cortisol was collected using cotton eye swabs; the swab was placed in the child’s cheek by the researcher until it was fully wet. Three swabs were collected and placed in a Salametrics tube. Saliva samples were frozen and stored at −20 °C until analysis. After thawing, salivettes were centrifuged at 3000 rpm for 5 min, which resulted in a clear supernatant of low viscosity. Salivary concentrations were measured using commercially available chemiluminescence immunoassay with high sensitivity (IBL International, Hamburg, Germany). Sample and reagent handling was semi-automated using a liquid handling robot (Genesis, Tecan, Switzerland) and quality control samples of low, medium, and high cortisol concentrations were run on each microtiter plate assayed. The intra and interassay coefficients for cortisol were both below 8%. The derived cortisol levels were winsorized and cortisol reactivity was assessed by calculating a difference score between the mean of the two baseline cortisol samples and the post-stressor sample.

Cortisol levels vary throughout the course of the day, however, as this investigation was part of a large-scale longitudinal study, where substantial number of participants were seen over a short period of time, it was necessary to assess during morning and afternoons, and hence unrealistic to conduct all the assessments at the same time of day. Time of first cortisol sample was at average 11:58 (SD 2:11 h) and ranged from 8:54 to 17:20. There was no association between cortisol reactivity and time of day of cortisol assessment, on the full sample (*r* = −.05, *p* = .390) nor in boys (*r* = −.03, *p* = .700) or girls (*r* = −.07, *p* = .389) separately. Steroid medication use is also known to affect cortisol levels. Information on current prescription and non-prescription medications usage was collected and medications were dichotomised into steroid-related versus non-steroid-related medication or no medication. Forty (14%) participants had used steroid-related medications within the last 2 weeks; 27 reported cortisol-based cream use, 12 inhaled steroid use and 1 oral tablet steroid use. There were no significant differences in cortisol reactivity between children who had used steroid medication and those who had not (*p* = .358 full sample, *p* = .506 girls, and *p* = .738 boys). Medication use significantly predicted higher baseline cortisol levels on the full sample (*t* = −1.98, df = 280, *p* = .048; no medication use mean = 7.04, medication use mean = 8.77) and in girls (*t* = −3.56, df = 135, *p* < .001; no medication use mean = 7.07, medication use mean = 12.74) but not in boys (*p* = .695). To account for any potential confounding effects on cortisol reactivity we ran a linear regression predicting cortisol reactivity from time of day and steroid medication use and used the residual score for the main analysis.

#### CU traits

CU traits were assessed by mother-report at 5 years using a combination of items from the APSD^30^, the CBCL^29^, and the Strengths and Difficulties Questionnaire (SDQ; 40). All items are rated on a three-point scale. Items were selected based on inclusion in CU traits measures in other studies^41–44^. We have previously created CU traits latent factor scores on this sample at ages 2.5, 3.5, and 5 years^45^ by subjecting items to exploratory and confirmatory factor analyses in MPlus^46^. The age 5 measure comprises 13 items which are listed in Supplementary Table S1 together with the factor loadings. The derived CU traits measure shows improved internal consistency (*α* = .83) compared to the APSD alone (*α* = .60) and partial strong factorial invariance by sex.

#### Aggression

Aggression was assessed by mother-report on a five-item physical aggression questionnaire at ages 5 and 7 years^47^. The questionnaire consists of five items previously shown to yield aggression scores with stability from ages 17 to 29 months^47^. Each item is rated on a three-point scale. The items were subjected to a confirmatory factor analysis in Mplus and a factor score was extracted for analysis.

#### Confounders

Deprivation was assessed using the indices of multiple deprivation (IMD; 30) based on UK postal codes in which a binary variable, with 1 = most deprived quintile of UK neighbours versus 0 = all other quintiles, was used for analysis. To account for the stratification, variables indicating whether the family was high-risk or low-risk allocation to the intensive sample were also included as covariates.

### Analysis plan

First, the age 7 years mother and teacher aggression items were modelled as a single latent variable using the gsem command in Stata version 14^48,49^. A factor score was extracted for all subsequent analysis. Bivariate associations were examined using Spearmans and polychoric correlations. The main analysis used multiple linear regression with predictors entered as a series of blocks using the nestreg command which provides a Wald test of whether the addition of each block produces a significant improvement in the model. The first block contained the confounding variables (including the two stratification variables) and the main effects of child sex, age 5 aggression and CU traits and cortisol reactivity was added in the second block. The two-way interaction term between CU traits and cortisol was added in the third block, and the three-way interaction at the third block, together with the other two-way interactions. All variables were standardised prior to creating interaction terms.

Interactions were further explored in two ways. First the margins command was used to test the association between CU traits and aggression at mean and 1 SD above and below the mean levels of cortisol reactivity. This was done in order to find out whether the interaction arose in accordance with the prediction that the association between CU traits and aggression will be greatest in the presence of low cortisol reactivity. Secondly, to address two potential limitations of the interaction, namely that it might be evident only in the presence of an increase in cortisol from baseline to post-stress, and that it may not be seen at the upper end of the CU traits distribution likely to be most relevant to clinical samples, we explored the linearity of the interaction of covariate *X* with moderator *M*. This was done by rewriting the usual regression of the form *E*[*y*] = *a* + *b*1*X* + *b*2*M* + *cXM* into the equivalent form *E*[*y*] = *a* + (*b*1 + *cM*)*X* + *b2M*. We then plotted the coefficient (*b*1 + *cM*) with its 95% confidence envelope that assumed linearity. To examine non-linearity we estimated group-specific estimates of this coefficient for different levels of the moderator variables, the groups being defined by deciles of cortisol reactivity and the levels being the median values of these groups. Since these group-wise estimates are highly variable, being estimated on small samples, the figure also displays a fractional polynomial smooth through them. To account for confounders, a model with the linear interaction and confounder main effects was first fitted, and the models required for the figure fitted to the adjusted aggression score obtained by subtracting the estimated effects of confounders.

We checked the distribution of the residuals from the analysis of the regression scores. Plots suggested modest skew but the Cook–Weisberg test was clearly significant (*p* < .001). Analyses were repeated with the three observations further than 3SD from the mean removed, and also with robust standard errors that are robust to heteroscedasticity. Both analyses left the pattern of significant effects unchanged. Since the analyses with robust standard errors are likely to be the most reproducible, it is these that we report. The regression models were also estimated with all variables entered sumulataneously and are presented in the Supplementary Tables 3 and 5. Power calculations for the original cohort were approved by the funder (MRC) following peer review.

## Results

### Computation of physical aggression outcome

The factor loadings for the mother and teacher aggression items on the single aggression latent variable are shown in Supplementary Table 2 for the extensive sample. A factor score was extracted for all subsequent analyses.

The descriptive statistics for the key study variables are presented in Table 1 for boys and girls separately. As can be seen in Table 1 mean cortisol levels were lower post-stress than at the baselines, with levels decreasing from baseline one to baseline two. In the full sample, 30% of children (*n* = 86) showed a rise from baseline to post-stress, with 29% of girls (*n* = 40) and 32% of boys (*n* = 46). Bivariate associations are presented in Table 2, for boys and girls separately. Cortisol reactivity showed no significant associations with CU traits or age 5 or 7 aggression. CU traits were associated with age 5 and age 7 aggression in both boys and girls. Mothers’ younger age at first pregnancy and deprivation were associated with increased aggression and CU traits, underlining the importance of controlling for these variables in subsequent analyses.

**Table 1:** Descriptive statistics for the key study variables for boys and girls separately

	Boys *N* = 145		Girls *N* = 138	
	Mean (SD)	Range	Mean (SD)	Range
*Age 7*				
Physical aggression (teacher and mother report)^a^	.68 (2.05)	−.79 to 7.22	−.18 (1.33)	−.79 to 5.31
*Age 5*				
Cortisol baseline 1	7.34 (4.19)	1.03–26.56	8.17 (5.88)	1.90–30.37
Cortisol baseline 2	6.49 (5.43)	.59–33.68	7.30 (6.94)	1.01–33.68
Cortisol post-stressor	5.67 (4.99)	.47–30.52	6.68 (5.87)	1.49–30.52
Aggression (mother report)^a^	.45 (.69)	−.03 to 1.81	.24 (.56)	−.03 to 1.81
CU traits^a^	.13 (.34)	−.51 to 1.20	.02 (.34)	−.51 to 1.04
*Confounding variables*				
Mothers age at pregnancy	27.30 (6.27)	18–51	27.66 (5.94)	18–41
Most deprived^b^: *n* (%)	51 (35.2)	0–1	56 (40.1)	0–1

^a^Factor scores extracted from a latent variable

^b^Most deprived = in highest national quintile for deprivation

**Table 2:** Bivariate associations between the key study variables by sex; boys on top diagonal and girls on bottom diagonal

	Age 7 agg.	Age 5 agg.	CU traits age 5	Cortisol reactivity age 5	Mother age	Most deprived	Preg. strat.	3.5 year strat.
Age 7 aggression		.52***	.40***	−.02	−.17*	.04	.03	.20*
Age 5 aggression	.23**		.43***	.02	−.14^†^	.04	−.01	.36***
CU traits, age 5	.31***	.42***		.08	−.19*	.14^†^	−.02	.35***
Cortisol reactivity, age 5	.12	.06	−.02		.10	.02	−.10	.03
Mother age at conception	−.12	−.06	−.05	.13		−.27***	−.08	−.20*
Most deprived^a^	−.05	.07	.04	−.12	−.33***		-.01	.09
Stratification in pregnancy	.08	.03	−.02	−.06	−.01	.03		−.38***
Stratification age 3.5 years	.04	.16^†^	.10	−.05	−.04	−.03	−.29***	
Mean (SD)	.26 (1.79)	.35 (.64)	.07 (.35)	−.02 (.95)	27.47 (6.10)	.38 (.49)	.61 (.76)	.20 (.40)

^†^*p* < .10; *p < .05; **p < .01; ***p < .001

^a^Most deprived = in highest national quintile for deprivation

### Multivariate analysis

**Fig. 1:**
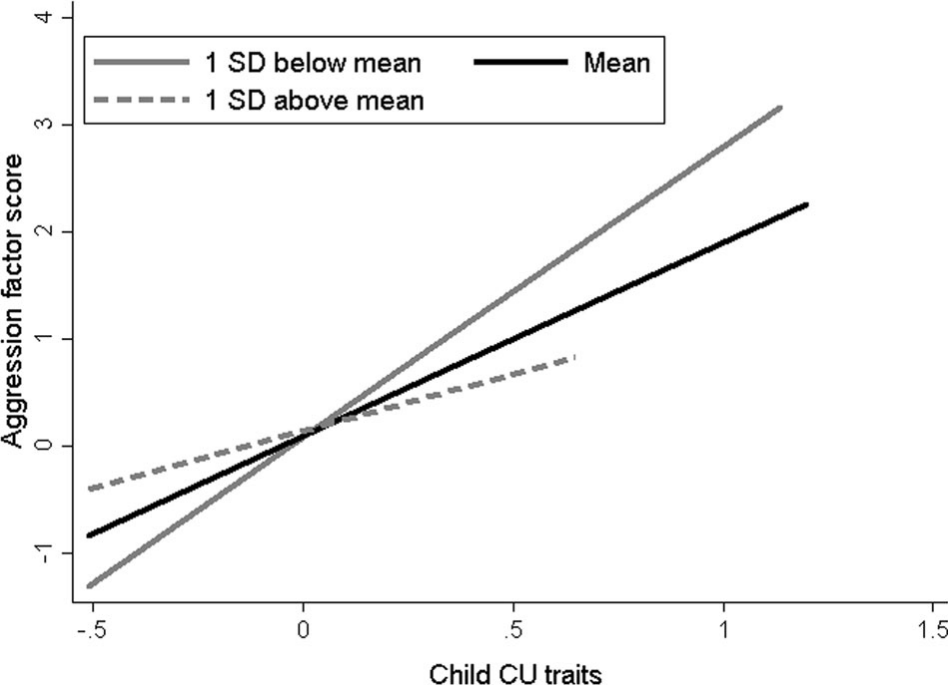
The prospective association between CU traits and aggression at ‘low’ (1 SD below mean), ‘medium’ (mean) and ‘high’ (1 SD above mean) cortisol reactivity

The results of the main analysis are presented in Table 3. CU traits at age 5 years predicted child aggression at age 7 years, controlling for age 5 years aggression, however there was no main effect of cortisol reactivity. The two-way interaction between CU traits and cortisol reactivity, introduced in the third block, was significant (*p* = .011). The effect of the interaction is shown in Fig. 1 contrasting associations between CU traits at age 5 and aggression at age 7 at low (1 SD below mean), medium (mean), and high levels of reactivity (1 SD above mean). The association between CU traits and child aggression was greatest in association with low cortisol reactivity (*β* = .50, SE = .12, *p* < .001) and progressively lessened at mean reactivity (*β* = .36, SE = .10, *p* < .001) and at high reactivity (*β* = .22, SE = .13, *p* = .084).

**Table 3:** Summary of linear regression model predicting age 7 aggression from age 5 cortisol reactivity, CU traits and child sex

	*β*	*p*
*Block 1*		
Mothers age	−.11	.056
Most deprived	−.07	.195
Sample stratification status: pregnancy stratum 1	−.01	.798
Sample stratification status: pregnancy stratum 2	.05	.490
Sample stratification status: 3.5 years	−.01	.881
Child sex^a^	−.14	.006
Age 5 aggression	.32	*p* < .001
Age 5 CU traits	.21	*p* < .001
	*F*(9, 274) = 8.98, *p* < .001, *R*^2^ = .27	
*Block 2*		
Cortisol reactivity	.01	.816
	*F*(1, 282) = .05, *p* = .816, *R*^2^ = .27, *R*^2^Δ = .00	
*Block 3*		
CU traits*Cortisol reactivity	−.11	.011
	*F*(1, 282) = 6.57, *p* = .011, *R*^2^ = .28, *R*^2^Δ = .01	
*Block 4*		
CU traits*Cortisol reactivity	−.37	.007
Child sex*Cortisol reactivity	.05	.758
Child sex*CU traits	−.24	.165
Child Sex*CU traits*Cortisol reactivity	.27	.041
	*F*(3, 280) = 2.07 *p* = .10, *R*^2^ = .29, *R*^2^Δ = .01	

^a^Child sex coded as 1 = male, 2 = female

The three-way interaction between sex, cortisol reactivity, and CU traits was significant (*p* = .041) reflecting that there was a two-way interaction in boys (*p* = .016) but not in girls (*p* = .799; the full model coefficients are presented in Supplementary Table 4 and Supplementary Figure 1 presents the interaction, for boys and girls seperately). In boys, CU traits significantly predicted aggression at low reactivity (*β* = .78, SE = .18, *p* < .001) and at mean reactivity (*β* = .49, SE = .15, *p* = .001) but not at high reactivity (*β* = .19, SE = .19, *p* = .276).

**Fig. 2:**
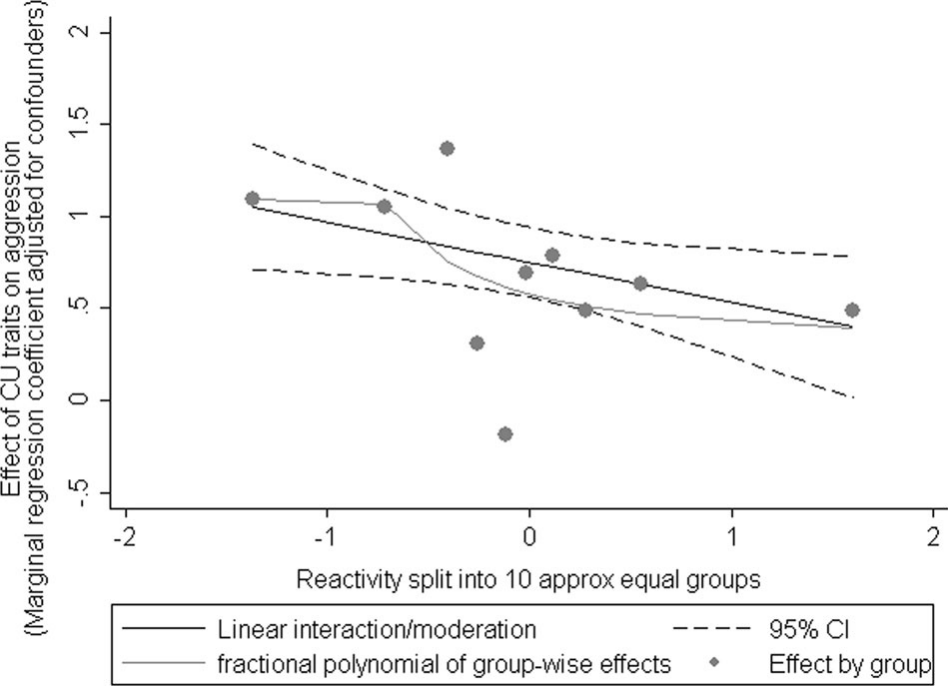
The effect of CU traits on aggression at 10 deciles of cortisol reactivity (adjusted for confounders)

Figure 2 plots the interaction at 10 deciles of cortisol reactivity. The figure indicates that the changing association between CU traits and aggression occurs across the distribution on reactivity and not among only those who showed a rise in cortisol after the stressor. Supplementary Figure 2 presents this plot in boys and girls separately; the boys plot largely mirrors that found on the full sample with the girls plot consistent with no moderation by cortisol reactivity. Supplementary Figures 3 and 4 plot the interaction at deciles of CU traits, on the whole sample and in boys and girls separately, respectively. The figures indicate that the interaction can be seen across the distribution of CU traits scores and is not restricted to high or low scorers.

## Discussion

In a longitudinal general population sample with cortisol reactivity to a social stressor, CU traits and child aggression assessed at age 5 years, the highest levels of aggression at age 7 years were predicted by the combination of high CU traits and low cortisol reactivity. This is consistent with the hypothesis put forward by Hawes et al.^11^. The two-way interaction was seen across the sample, but the effect was further modified by sex of child. The three-way interaction arose because in girls there was an association between CU traits and later aggression that was similar at all levels of cortisol reactivity, while in boys the association was markedly different at low and high levels. This effect of CU traits and cortisol reactivity at age 5 years on aggression at 7 years remained after controlling for child aggression at age 5 years.

Although the prediction of age 7 aggression from age 5 CU traits was not the main focus of the study, the finding of an association over and above the continuity between age 5 and age 7 aggression provided further support for a continuing effect of CU traits even after age 5 when child aggression has commonly become established. This is a well replicated effect in older children, consistent with growing evidence in young children^6,50^. The lack of main effect of cortisol reactivity on child aggression from age 5 to age 7 is consistent with previous findings.^16^.

While the significant interaction between CU traits and cortisol reactivity was seen across the sample as a whole, it was driven by the effect in boys, and was not seen at all in girls. This sex difference has been reported in previous studies of HPA-axis activity outlined earlier^5,7,26,37^. It is also consistent with other findings of physiogical reactivity and externalising problems in children. In relation to respiratory sinus arrhythmia (RSA), reductions to a stressor, used as an index of vagal reactivity, several studies have found externalising symptoms associated with decreased vagal reactivity in boys but increased reactivity in girls^6–8,10^. Twin studies of the aeteology of both CU traits and conduct problems have identified sex differences, in particular a greater shared environment influence for girls than boys^51,52^. Collectively this evidence suggests that there may be distinct aeteological pathways to the development of antisocial behaviour in boys and girls.

The study had a number of strengths. This was a prospective study of a consecutive sample from an antenatal clinic serving a defined geographical area. The subsample was created by stratifying for psychosocial risk during pregnancy and child symptoms at age 3.5 years and inclusion of the sample stratification factors in the models allowed generalisations to be made to the general population. Child CU traits and cortisol reactivity were assessed at age 5 and aggression was assessed at ages 5 and 7. Both teacher and mother report of aggression were collected at age 7 and combined to produce a single latent variable outcome. This created a more robust aggression outcome which sampled behaviour in multiple domains, reduced the risk of inferential errors from multiple testing and also helped to reduce the effect of common method variance on the reporting of CU traits and aggression.

A key limitation of the study is that the experimental stressor for cortisol reactivity did not lead to an overall rise in cortisol levels. This is a widely reported finding in studies of cortisol reactivity in young children^53^. Within the context of a longitudinal study where retention of participants over a long period of time is paramount, higher levels of stress may have meant that children would not have wanted to remain in the study. In this sample we observed an overall decrease in cortisol levels from the first to second baseline and then to the post-stressor samples, consistent with an initial rise in response to arrival in the lab which reduced throughout the testing session. This would support the possibility that the response to the planned stressor was superimposed on falling cortisol following the first unplanned stressor on arrival at the lab. Whether or not variations in rates of fall following the planned stressor provide valid measures of an individual’s reactivity, in the same way as variations in increases, is not known. As far as we are aware we are the first to have set out to examine this by assessing whether the cortisol reactivity by CU traits interaction varied across the distribution of reactivity scores, and in particular whether the effect was confined to the subgroup of children showing a pre-post stressor rise. There was convincing evidence that this was not the case supporting the validity of pre-post differences in cortisol even in the absence of an overall rise. This is consistent with findings from a number of other studies of young children that have yielded informative findings in the absence of overall effects on cortisol levels^54–57^, including prospective associations between cortisol reactivity to a lab stressor at age 3 years and externalising and internalising symptoms at age 6 years^54^.

The need to assess large numbers of children over a short period of time also meant that cortisol collections were not completed at the same time of day for all participants. However, this was accounted for by a correction for time of day in all analyses by creating a residualised cortisol score. The sample was recruited from a defined geographical area with a wide range of socioecomic conditions, but with very few non-white families, so the findings may not be not generalisable to other ethnic groups. This study used a community sample to investigate the processes involved in the translation of CU traits to aggressive behaviour, with only a minority of the sample showing clinically significant behavioural problems, and so it cannot be assumed that the findings generalise to clinical populations. However, we showed that the interaction between CU traits and cortisol reactivity could be seen across the distribution on CU traits scores, providing first evidence that these processes operate similarly in children with both high and low CU traits scores. Finally, we used a brief physical aggression measure which did not distinguish different forms of aggressive behaviour when developmental models of CU traits have placed emphasis on their role in proactive aggression^57^.

The findings reported here were based on the hypothesis that both reduced amygdala reactivity to others’ emotions and hence lower empathy, and reduced arousal and hence reduced inhibition, would jointly contribute to risk for aggression. While the interactions that we found may reflect such a synergy, other explanations are possible. Nevertheless they suggest that further investigation of the role of amygdala reactivity together with HPA axis reactivity would add to our understanding of mechanisms in male aggression. Whether or not there are different mechanisms in girls also requires further study.

## Supplementary information

Supplementary Figure 1. The prospective association between CU traits and aggression at ‘low’ (1 SD below mean), ‘medium’ (mean) and ‘high’ (1 SD above mean) cortisol reactivity in boys and girls

Supplementary Figure 2. The effect of CU traits on aggression at approximate deciles of cortisol reactivity in boys and girls separately (adjusted for confounders)

Supplementary Figure 3. The effect of cortisol reactivity on aggression at approximate deciles of CU traits (adjusted for confounders)

Supplementary Figure 4. The effect of cortisol reactivity on aggression at approximate deciles of CU traits in boys and girls separately (adjusted for confounders)

Supplementary Table 1: Standardised factor loadings for the age 5 years CU traits measure

Supplementary Table 2: Unstandardised factor loadings for the teacher and mother reported aggression items

Supplementary Table 3: Full model coefficients for the linear regression model including the three-way interaction with child sex predicting aggression

Supplementary Table 4: Linear regression with robust standard errors predicting age 7 aggression from age 5 cortisol reactivity and CU traits in boys and girls separately

Supplementary Table 5: Full model coefficients for the two linear regression models estimated in boys and girls separately
