# Supplementary figures and images for: Callous-unemotional traits, low cortisol reactivity and physical aggression in children: findings from the Wirral Child Health and Development Study
Source: Transl Psychiatry. 2019 Feb 11;9:79. doi: 10.1038/s41398-019-0406-9 (PMC6370839; doi:10.1038/s41398-019-0406-9)

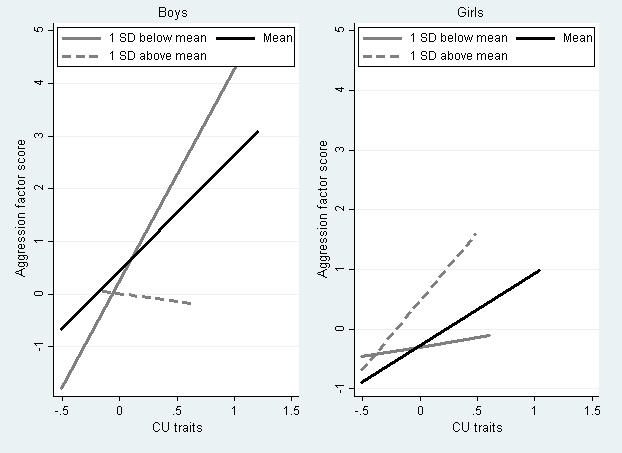

Supplement: Supplementary file 1 — Supplementary Figure 1. The prospective association between CU traits and aggression at ‘low’ (1 SD below mean), ‘medium’ (mean) and ‘high’ (1 SD above mean) cortisol reactivity in boys and girls [file 41398_2019_406_MOESM1_ESM.tif]

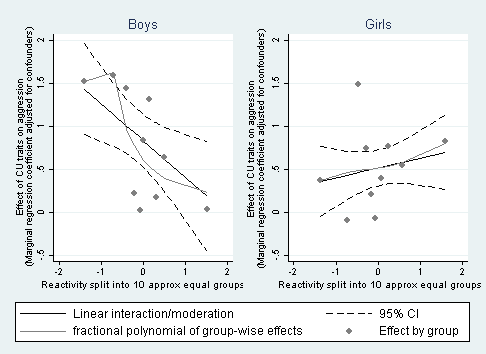

Supplement: Supplementary file 2 — Supplementary Figure 2. The effect of CU traits on aggression at approximate deciles of cortisol reactivity in boys and girls separately (adjusted for confounders) [file 41398_2019_406_MOESM2_ESM.tif]

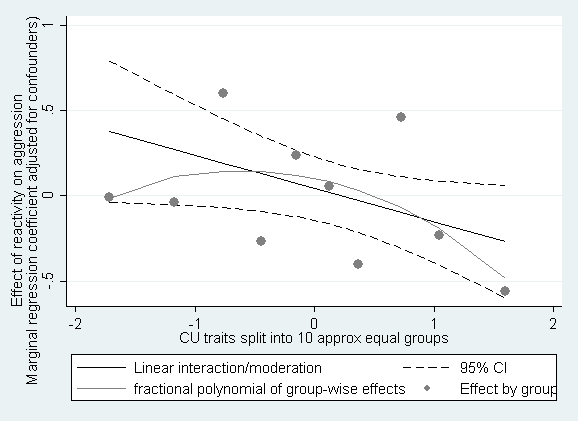

Supplement: Supplementary file 3 — Supplementary Figure 3. The effect of cortisol reactivity on aggression at approximate deciles of CU traits (adjusted for confounders) [file 41398_2019_406_MOESM3_ESM.tif]

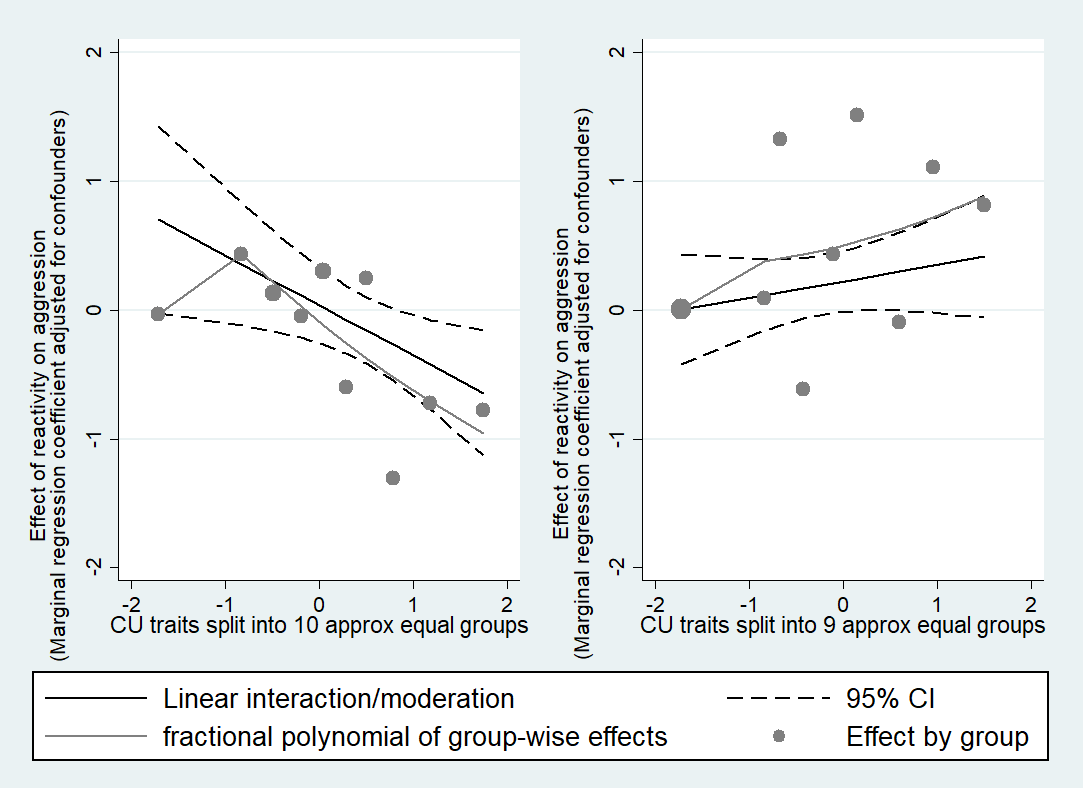

Supplement: Supplementary file 4 — Supplementary Figure 4. The effect of cortisol reactivity on aggression at approximate deciles of CU traits in boys and girls separately (adjusted for confounders) [file 41398_2019_406_MOESM4_ESM.tif]
