# Supplementary material for: Callous-unemotional traits, low cortisol reactivity and physical aggression in children: findings from the Wirral Child Health and Development Study
Source: Transl Psychiatry. 2019 Feb 11;9:79. doi: 10.1038/s41398-019-0406-9 (PMC6370839; doi:10.1038/s41398-019-0406-9)
Supplement: Supplementary file 5 — Supplementary Table 1: Standardised factor loadings for the age 5 years CU traits measure [file 41398_2019_406_MOESM5_ESM.docx]

Supplementary Table 1: Standardised factor loadings for the age 5 years CU traits measure

| Age 5 CU traits items | Factor loading |
| --- | --- |
| APSD 1: Concerned about the feelings of others (R) | .47 |
| APSD 3: Is good at keeping promises (R) | .47 |
| APSD 4: Feels bad or guilty when he/she does something wrong (R) | .62 |
| APSD 5: Keeps the same friends (R) | .61 |
| CBCL 14. Cruel to animals | .60 |
| CBCL 58: Punishment doesn’t change his/her behavior | .72 |
| CBCL 67: Seems unresponsive to affection | .77 |
| CBCL 70: Shows little affection toward people | .84 |
| SDQ 1: Considerate of other people’s feelings (R) | .75 |
| SDQ 4: Shares readily with other children (R) | .53 |
| SDQ 9: Helpful if someone is hurt, upset or feelings ill (R) | .57 |
| SDQ 17: Kind to younger children (R) | .60 |
| SDQ 20: Often volunteers to help others (R) | .46 |

*Note.* CBCL = Child Behavior Checklist (CBCL), APSD = Anti-Social Process Screening Device, BITSEA = Brief Infant Toddler Social and Emotional Assessment (BITSEA), SDQ = Strengths and Difficulties Questionnaire (SDQ) .
