# Supplementary material for: Callous-unemotional traits, low cortisol reactivity and physical aggression in children: findings from the Wirral Child Health and Development Study
Source: Transl Psychiatry. 2019 Feb 11;9:79. doi: 10.1038/s41398-019-0406-9 (PMC6370839; doi:10.1038/s41398-019-0406-9)
Supplement: Supplementary file 6 — Supplementary Table 2: Unstandardised factor loadings for the teacher and mother reported aggression items [file 41398_2019_406_MOESM6_ESM.docx]

Supplementary Table 2: Unstandardised factor loadings for the teacher and mother reported aggression items

|  | Unstandardised factor loading mother report | Unstandardised factor loading teacher report |
| --- | --- | --- |
| Bites other children | 1.11*** | 1.21** |
| Kicks other children | .90*** | 2.40* |
| Hits other children | .66*** | 1.54*** |
| Gets in many fights | .65** | 1.00*** |
| Physically attacks others | .55*** | 1.90** |
