# Supplementary material for: Callous-unemotional traits, low cortisol reactivity and physical aggression in children: findings from the Wirral Child Health and Development Study
Source: Transl Psychiatry. 2019 Feb 11;9:79. doi: 10.1038/s41398-019-0406-9 (PMC6370839; doi:10.1038/s41398-019-0406-9)
Supplement: Supplementary file 7 — Supplementary Table 3: Full model coefficients for the linear regression model including the three-way interaction with child sex predicting aggression [file 41398_2019_406_MOESM7_ESM.docx]

|  | β | p |
| --- | --- | --- |
| Mothers age | -.10 | .103 |
| Most deprived | -.06 | .251 |
| Sample stratification status: pregnancy stratum 1 | -.01 | .879 |
| Sample stratification status: pregnancy stratum 2 | .04 | .543 |
| Sample stratification status: 3.5 years | -.01 | .982 |
| Child Sex | -.15 | .004 |
| Age 5 aggression | .29 | p<.001 |
| CU traits | .44 | .020 |
| Cortisol reactivity | -.05 | .756 |
| Cortisol reactivity * CU traits | -.34 | .007 |
| Cortisol reactivity * Sex | -.24 | .165 |
| CU traits * Sex | .11 | .472 |
| Cortisol reactivity * CU traits * Sex | .26 | .041 |

Supplementary Table 3: Full model coefficients for the linear regression model including the three-way interaction with child sex predicting aggression
