# Supplementary material for: Callous-unemotional traits, low cortisol reactivity and physical aggression in children: findings from the Wirral Child Health and Development Study
Source: Transl Psychiatry. 2019 Feb 11;9:79. doi: 10.1038/s41398-019-0406-9 (PMC6370839; doi:10.1038/s41398-019-0406-9)
Supplement: Supplementary file 8 — Supplementary Table 4: Linear regression with robust standard errors predicting age 7 aggression from age 5 cortisol reactivity and CU traits in boys and girls separately [file 41398_2019_406_MOESM8_ESM.docx]

Supplementary Table 4: Linear regression with robust standard errors predicting age 7 aggression from age 5 cortisol reactivity and CU traits in boys and girls separately.

|  | **Boys** | | **Girls** | |
| --- | --- | --- | --- | --- |
|  | **β** | **p** | **β** | **p** |
| *Block 1* |  |  |  |  |
| Mothers age | -.09 | .254 | -.15 | .078 |
| Most deprived | -.03 | .700 | -.12 | .137 |
| Sample stratification status: pregnancy stratum 1 | .05 | .445 | -.06 | .416 |
| Sample stratification status: pregnancy stratum 2 | -.01 | .973 | .12 | .288 |
| Sample stratification status: 3.5 years | -.02 | .793 | -.02 | .766 |
| Age 5 aggression | .43 | P<.001 | .12 | .211 |
| CU traits | .22 | .008 | .28 | .002 |
|  | *F*(7, 138) = 8.14 *p* <.001. *R^2^* .31 | | *F*(7, 131) = 2.00 *p* = .059. *R^2^* .15 | |
| *Block 2* |  | |  | |
| Cortisol reactivity | -.05 | .487 | .13 | .157 |
|  | Overall *R*^2^ .31. Block F=.059 (1, 144) *p* = .442, *R*^2^ Δ = .00 | | Overall *R*^2^ .16. Block F(1, 137) = 1.99 *p* = .157 *R*^2^Δ = .01 | |
| *Block 3* |  |  |  |  |
| CU traits * Cortisol reactivity | -.12 | .016 | .02 | .799 |
|  | Overall *R*^2^ .31. Block F(1, 144) = 5.89, p = .016, *R*^2^Δ = .01 | | Overall *R*^2^ .16. Block F(1, 137) = .06 p =.799. *R*^2^Δ = .00 | |
