# Supplementary material for: Callous-unemotional traits, low cortisol reactivity and physical aggression in children: findings from the Wirral Child Health and Development Study
Source: Transl Psychiatry. 2019 Feb 11;9:79. doi: 10.1038/s41398-019-0406-9 (PMC6370839; doi:10.1038/s41398-019-0406-9)
Supplement: Supplementary file 9 — Supplementary Table 5: Full model coefficients for the two linear regression models estimated in boys and girls separately [file 41398_2019_406_MOESM9_ESM.docx]

|  | Boys | | Girls | |
| --- | --- | --- | --- | --- |
|  | β | p | β | p |
| Mothers age | -.08 | .361 | -.17 | .078 |
| Most deprived | -.02 | .754 | -.11 | .197 |
| Sample stratification status: pregnancy stratum 1 | .04 | .556 | -.05 | .514 |
| Sample stratification status: pregnancy stratum 2 | .04 | .972 | .12 | .279 |
| Sample stratification status: 3.5 years | -.01 | .926 | -.01 | .938 |
| Age 5 aggression | .40 | p<.001 | .11 | .306 |
| CU traits | .21 | .010 | .28 | .002 |
| Cortisol reactivity | .01 | .887 | .14 | .169 |
| Cortisol reactivity * CU traits | -.12 | .016 | .03 | .750 |

Supplementary Table 5: Full model coefficients for the two linear regression models estimated in boys and girls separately
